# The complete chloroplast genome of *Viola grypoceras* (Violaceae)

**DOI:** 10.1080/23802359.2022.2160216

**Published:** 2023-01-02

**Authors:** Jin Hee Park, Minjee Lee, Yi Lee, Jungho Lee

**Affiliations:** a Nakdonggang National Institute of Biological Resources, Sangju, Republic of Korea; b Green Plant Institute, Yongin, Republic of Korea; c Department of Industrial Plant Science & Technology, Chungbuk National University, Cheongju, Republic of Korea

**Keywords:** Chloroplast genome, next-generation sequencing, *Viola grypoceras*

## Abstract

We constructed and characterized the chloroplast genome of *Viola grypoceras* via *de novo* assembly of Illumina data. The complete circular chloroplast genome is 158,357 bp long and contains four parts: a large single-copy (LSC) region of 86,764 bp, a small single-copy (SSC) region of 17,345 bp, and two inverted-repeat regions (IRa and IRb) of 27,124 bp each. Genome annotation predicted that this genome harbors 111 genes, comprising 77 protein-coding genes, 30 transfer RNA (tRNA) genes, and four ribosomal RNA (rRNA) genes. Phylogenetic analysis demonstrated that *V. grypoceras* shares a close systematic relationship with *V. mirabilis* and *V. websteri* by forming a basal clade in the genus *Viola*.

The violet *Viola grypoceras* Gray 1857 is a Northeast Asian plant species, distributed in Korea, Japan, Taiwan, and mainland China (Akiyama et al. 2002; Chen et al. 2007; Lee and Yoo 2020). In Korea, this species occupies the southernmost parts of the Korean peninsula and Jeju Island. *V. grypoceras* plants have aerial stems and flowers with purplish to white petals with purple stripes. Plastid genomic information is available for only a few *Viola* species with aerial stems (Cheon et al. 2019; Kwak 2021). To explore the relationships among the Korean *Viola* species, especially caulescent species, the whole chloroplast genome sequence was studied based on next-generation sequencing (NGS).

In the present study, we generated the complete chloroplast genome sequence of *V. grypoceras*. Fresh leaf samples were collected for DNA extraction on the banks of Gangjeong-cheon stream in Jeju Island, South Korea (33°28′2.25″N, 126°30′56.55″E). A dried plant specimen was deposited in the Herbarium of the Nakdonggang National Institute of Biological Resources (NNH) under voucher number NNIBRVP90496 (https://fbp.nnibr.re.kr/portal/; contact: Jin Hee Park; parkjh23@nnibr.re.kr). Total genomic DNA was extracted from the leaf tissue using a DNeasy Plant Mini Kit (Qiagen, Valencia, CA). The isolated genomic DNA was used to construct a paired-end library (PE) with a mean insert size of 500 bp by Theragen Bio (Suwon, South Korea), followed by sequencing on an Illumina HiSeq 2500 platform. A total of 25.37 Gb of 150-bp PE reads were obtained by Illumina HiSeq NGS and assembled using CLC Genomics Workbench (ver. 8.05 CLC Inc., Aarhus, Denmark) (Jeong et al. 2014). The chloroplast genome structure was verified using long PCR and Sanger sequencing (Lee et al. 2015). The assembled structure and the genes in the complete chloroplast genome were annotated using Sequin and were manually curated based on BLAST searches. The annotated genome was deposited in GenBank (accession no. OM055663).

The complete plastid genome of *V. grypoceras* is a quadripartite circular structure of 158,357 bp in length with a GC content of 36.2%. The genome is composed of four distinct regions: a large single-copy region of 86,764 bp, a small single-copy region of 17,345 bp, and a pair of identical inverted-repeat regions (IRa and IRb) of 27,124 bp each. We annotated 111 genes in the *V. grypoceras* chloroplast genome, which consists of 77 protein-coding genes, 30 transfer RNA (tRNA) genes, and four ribosomal RNA (rRNA) genes. The *V. grypoceras* chloroplast genome contains 20 introns, with one group I intron and 19 group II introns. The group I intron is present in the gene *trnL-UAA* (Besendahl et al. 2000). Nineteen group II introns are distributed in 16 genes. Three genes including *ycf3*, *clpP*, and *rps12* contain two group II introns. The first intron of *rps12* is a trans-splicing intron (Hildebrand et al. 1988; Lee et al. 2015). Twelve genes contain a single group II intron: *ndhA*, *ndhB*, *petB*, *petD*, *rpl2*, *rpl16*, *rpoC1*, *trnA-UGC*, *trnG-UCC*, *trnI-GAU*, *trnK-UUU*, and *trnV-UAC*. However, the chloroplast genomes of all *Viola* species including *V. grypoceras* lack the *rps16* gene and its group II intron, which is rarely absent in angiosperm plastid genomes (Alqahtani and Jansen 2021).

We compared seven genes (*psaA*, *psaB*, *psbA*, *psbB*, *psbC*, *psbD*, and *rbcL*) in algae (Jeong et al. 2014) and angiosperms (Hong et al. 2017) to examine the phylogenetic relationships among *Viola* species. We inferred the evolutionary history of these species using the maximum-likelihood method and General Time Reversible model (Nei and Kumar 2000) with 1000 bootstrap replications in MEGA X (Kumar et al. 2018).

Twelve *Viola* species formed three major clades in the phylogenetic tree, with 99–100% bootstrap support, using *Passiflora miniata* of the Passifloraceae and *Salix bicolor* of the Salicaceae as the outgroups (Figure 1). *V. grypoceras* belongs to the basal-most clade in the tree, along with *V. mirabilis* and *V. websteri*. Seven *Viola* species without aerial stems form a derived clade. Two caulescent species, *V. raddeana* and *V. verecunda*, were placed between the two clades. As shown in Figure 1, five *Viola* species with aerial stems were placed in basal lineages of the genus *Viola*. The phylogenetic tree places *V. grypoceras* close to *V. mirabilis.* Further investigation of the caulescent *Viola* taxa at the interspecies and intraspecies levels would further our understanding of the relationships of *Viola* species.

**Figure 1.**
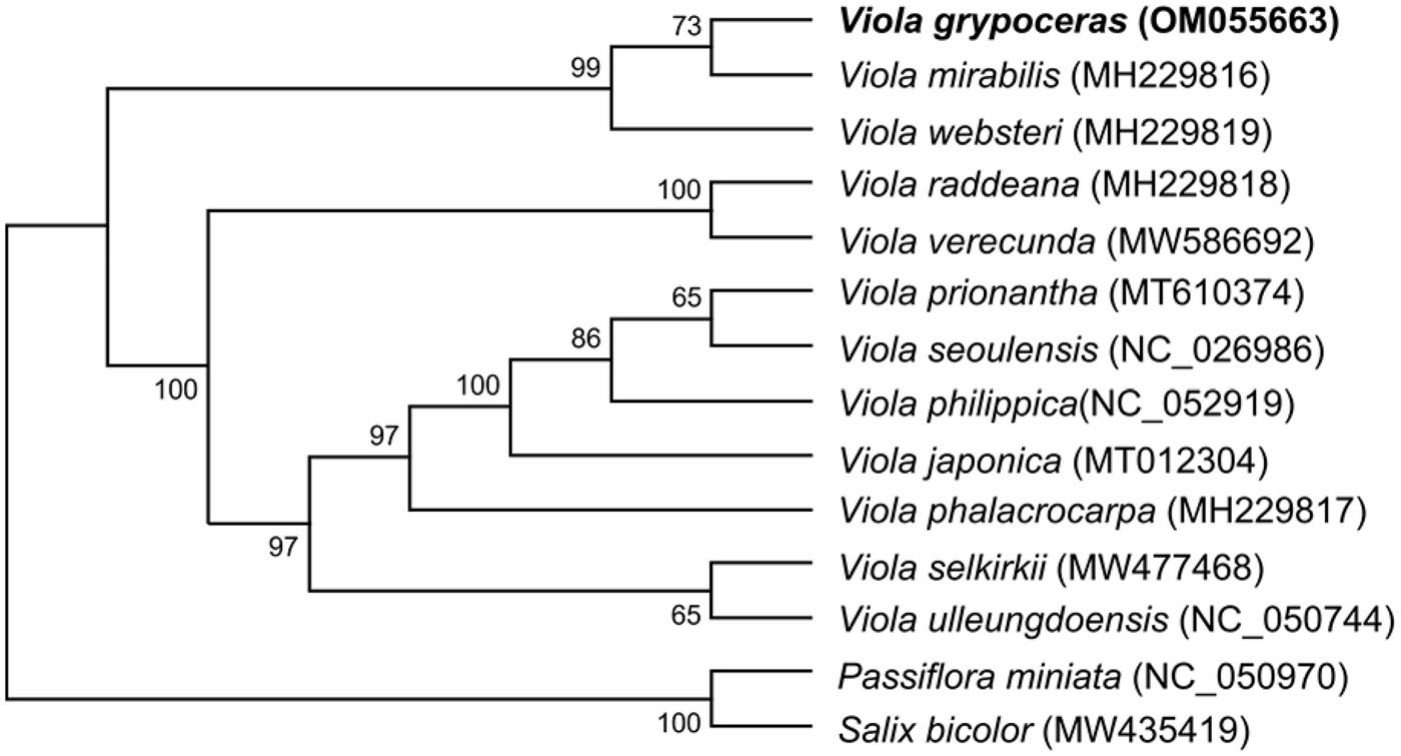
Maximum-likelihood phylogenetic tree based on seven chloroplast genes (*psaA*, *psaB*, *psbA*, *psbB*, *psbC*, *psbD*, and *rbcL*) from 14 species in the order Malpighiales. The Passifloraceae and Salicaceae families were used as the outgroups for Violaceae. Numbers at each node represent the bootstrap values for 1000 replicates.

## Data Availability

The genome sequence data that support the findings of this study are openly available in GenBank of NCBI at https://www.ncbi.nlm.nih.gov under the accession no. OM055663. The associated ‘BioProject’, ‘SRA’, and ‘Bio-Sample’ numbers are PRJNA816282, SRR18329144, and SAMN26665741, respectively.

## References

[CIT0001] Akiyama S, Ohba H, Tabuchi S. 2002. Violaceae. In: Iwatsuki K, Boufford DE, Ohba H, editors. Flora of Japan. Vol. c. Tokyo: Kodansha Ltd.; p. 161–190.

[CIT0002] Alqahtani AA, Jansen RK. 2021. The evolutionary fate of *rpl32* and *rps16* losses in the *Euphorbia schimperi* (Euphorbiaceae) plastome. Sci Rep. 11(1):7466. 33811236 10.1038/s41598-021-86820-z PMC8018952

[CIT0003] Besendahl A, Qiu Y-L, Lee J, Palmer JD, Bhattacharya D. 2000. The cyanobacterial origin and vertical transmission of the plastid tRNA^LEU^ group-I intron. Curr Genet. 37(1):12–23. 10672439 10.1007/s002940050002

[CIT0004] Chen Y, Yang Q, Ohba H, Nikitin VV. 2007. *Viola* L. In Wu ZY, Raven PH, Hong DY, editors. Flora of China. Vol. 13. Beijing: Science Press; p. 74–111.

[CIT0005] Cheon KS, Kim KA, Kwak M, Lee B, Yoo KO. 2019. The complete chloroplast genome sequences of four *Viola* species (Violaceae) and comparative analyses with its congeneric species. PLOS One. 14(3):e0214162. 30893374 10.1371/journal.pone.0214162 PMC6426196

[CIT0006] Gray A. 1857. List of dried plants collected in Japan, by S. Wells Williams, ESQ., and Dr. James Morrow. In: Perry MC, editor. Narrative of the expedition of an American Squadron to the China Seas and Japan, performed in the years 1852, 1853, and 1854. Under the command of Commodore M. C. Perry, United States Navy by order of the Government of the United States. Washington: A.O.P. Nicholson, Printer; p. 305–332.

[CIT0007] Hildebrand M, Hallick RB, Passavant CW, Bourque DP. 1988. Trans-splicing in chloroplasts: the rps12 loci of *Nicotiana tabacum*. Proc Natl Acad Sci U S A. 85(2):372–376. 3422433 10.1073/pnas.85.2.372 PMC279550

[CIT0008] Hong CP, Park J, Lee Y, Lee M, Park SG, Uhm Y, Lee J, Kim CK. 2017. accD nuclear transfer of *Platycodon grandiflorum* and the plastid of early Campanulaceae. BMC Genomics. 18(1):607. 28800729 10.1186/s12864-017-4014-x PMC5553655

[CIT0009] Jeong H, Lim JM, Park J, Sim YM, Choi HG, Lee J, Jeong WJ. 2014. Plastid and mitochondrion genomic sequences from Arctic *Chlorella* sp. ArM0029B. BMC Genomics. 15:286. 24735464 10.1186/1471-2164-15-286 PMC4023601

[CIT0010] Kumar S, Stecher G, Li M, Knyaz C, Tamura K. 2018. MEGA X: molecular evolutionary genetics analysis across computing platforms. Mol Biol Evol. 35(6):1547–1549. 29722887 10.1093/molbev/msy096 PMC5967553

[CIT0011] Kwak M. 2021. The complete chloroplast genome sequence of *Viola verecunda* (Violaceae). Mitochondrial DNA B. 6(12):3409–3410. 10.1080/23802359.2021.1997102 PMC8604497 34805517

[CIT0012] Lee M, Park J, Lee H, Sohn S-H, Lee J. 2015. Complete chloroplast genomic sequence of *Citrus platymamma* determined by combined analysis of Sanger and NGS data. Hortic Environ Biotechnol. 56(5):704–711.

[CIT0013] Lee WT, Yoo KO. 2020. Violaceae Batsch. In: Flora of Korea Editorial Committee , editor. Flora of Korea. Vol. 4a. Incheon, South Korea: National Institute of Biological Resources; p. 45–64.

[CIT0014] Nei M, Kumar S. 2000. Molecular evolution and phylogenetics. Oxford (UK): Oxford University Press; p. 333.

